# Retrotransposons shape species-specific embryonic stem cell gene expression

**DOI:** 10.1186/s12977-015-0173-5

**Published:** 2015-05-29

**Authors:** Luisa Robbez-Masson, Helen M Rowe

**Affiliations:** Division of Infection and Immunity, Medical Research Council Centre for Medical Molecular Virology, University College London, 90 Gower Street, London, WC1E 6BT UK

## Abstract

Over half of our genome is composed of retrotransposons, which are mobile elements that can readily amplify their copy number by replicating through an RNA intermediate. Most of these elements are no longer mobile but still contain regulatory sequences that can serve as promoters, enhancers or repressors for cellular genes. Despite dominating our genetic content, little is known about the precise functions of retrotransposons, which include both endogenous retroviruses (ERVs) and non-LTR elements like long interspersed nuclear element 1 (LINE-1). However, a few recent cutting-edge publications have illustrated how retrotransposons shape species-specific stem cell gene expression by two opposing mechanisms, involving their recruitment of stem cell-enriched transcription factors (TFs): firstly, they can activate expression of genes linked to naïve pluripotency, and secondly, they can induce repression of proximal genes. The paradox that different retrotransposons are active or silent in embryonic stem cells (ESCs) can be explained by differences between retrotransposon families, between individual copies within the same family, and between subpopulations of ESCs. Since they have coevolved with their host genomes, some of them have been co-opted to perform species-specific beneficial functions, while others have been implicated in genetic disease. In this review, we will discuss retrotransposon functions in ESCs, focusing on recent mechanistic advances of how HERV-H has been adopted to preserve human naïve pluripotency and how particular LINE-1, SVA and ERV family members recruit species-specific transcriptional repressors. This review highlights the fine balance between activation and repression of retrotransposons that exists to harness their ability to drive evolution, while minimizing the risk they pose to genome integrity.

## Part I: Retrotransposons are active in ESCs

### Retrotransposon activity fluctuates between distinct embryonic cell states

Retrotransposons, which may encompass over two-thirds of the human genome [1] have the potential to cause insertional mutagenesis or transcriptional perturbation prompting their epigenetic silencing during early embryonic development. Retrotransposons are then assumed to remain inactive throughout the organism’s adult life through their collective DNA methylation and their aberrant activation has been associated with cancer and autoimmune disorders [2, 3]. However, it has long been known that these elements also play normal roles in development, particularly in the placenta. A series of papers have now revealed that subsets of retrotransposons are expressed within mouse and human embryonic stem cell (ESC) cultures. The roles that these retrotransposons may play in development relate to their distinct expression patterns between different in vitro subpopulations of ESCs that are described below.

Embryonic stem cells are characterised by their capacity for self-renewal and pluripotency. They constitute a category of primary cells isolated from the inner cell mass (ICM) of blastocysts, which are early-stage pre-implantation embryos, and these cells can form tightly aggregated, three-dimensional colonies in culture. ESCs have been used as model systems to study the pluripotent state and the conditions required for cellular differentiation. Indeed, ESCs possess the remarkable capacity to self-renew and differentiate into cells of all three germ layers of the embryo (endoderm, mesoderm and ectoderm) including the germ line in vitro, or in vivo when ESCs are injected into blastocysts leading to the generation of chimeric mice [4].

Cultures of mouse ESCs display a great degree of intercellular heterogeneity, where cells can exhibit different states of pluripotency (as shown by their different gene expression profiles [5]). Cells within these cultures are dynamic, shuttling between a ground state termed “naïve” and a more committed “primed” state [6–8]. Hallmarks of naïve stem cells include high and stable expression of core pluripotency-associated TFs such as NANOG, OCT4 (POU5F1), the active *Oct4* distal enhancer, REX1, and SOX2. Moreover, these cells express key naïve (or ground-state) defining TFs, such as LBP9 and KLF4. Naïve cells also display global DNA hypomethylation, an absence of chromosome X inactivation and a reduced concentration of H3K27me3 repressive histone marks on the main developmental genes [7, 9]. Such ground state ESCs can be maintained in two-inhibitor (2i) medium supplemented with Leukaemia inhibitory factor (LIF). 2i culture conditions involve the addition of two specific small molecule inhibitors directed to the MAPK/ERK and GSK3 pathways. Since these pathways are both stimulated by LIF and can trigger cell differentiation and impair ESCs self-renewal, they both require inhibition [8]. It has recently been shown that a subpopulation of mouse ESCs express the ERV, MERV-L as well as a pool of transcripts normally expressed in two-cell (2-cell)-stage embryos, whereas they do not express classical pluripotency-associated factors like OCT4. Remarkably when isolated, these cells have totipotent potential instead of only pluripotent potential, which means that they can differentiate into extra-embryonic tissues (placenta) as well as into all three germ layers [10]. This is in line with previous data showing MERV-L to be highly expressed in 2-cell stage mouse embryos [11] before being repressed at the blastocyst stage.

In contrast to mouse ESCs, human ESC lines that have been derived, more closely resemble mouse epiblast stem cells (EpiSCs), a more advanced stage of development of the post-implantation embryo [12]. A lot of studies have focused on trying to “reset” human ESC cultures to a more naïve state of pluripotency in order to study this earlier stage of development. So far, no consensus has been established in terms of the optimum conditions for naïve human pluripotent cell culture, although three main protocols for generating these cells have recently been developed [13–15]. Some strategies require the use of different cocktails of small molecules inhibitors and cytokines to stimulate the expression of naïve pluripotency proteins such as LBP9 [13, 15, 16], whereas other approaches employ the direct introduction of pluripotency factors as transgenes to achieve the same effect (NANOG and KLF2 transgene expression coupled to ERK inhibition) [14].

**Figure 1:**
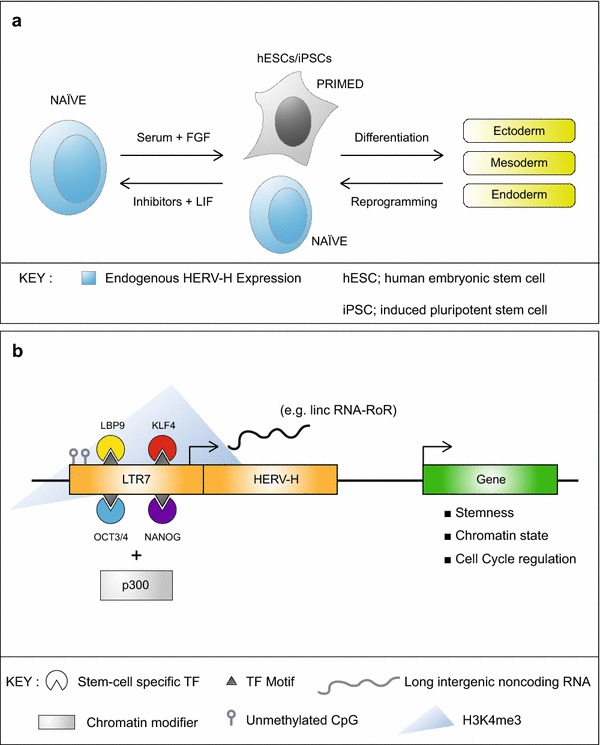
HERV-H activity overlaps with ground state pluripotency. **a** HERV-H expression is thought to define naïve-like stem cells. **b** Mechanism of HERV-H regulation of stem cell gene expression.

However, it has recently been shown that a natural population of naïve-like cells exists in human ESC cultures and transiently during reprogramming, and these cells can be isolated simply by using a GFP reporter driven by a HERV-H promoter, since they express this primate-specific retrovirus ([17–19] and see Figure 1a). This would obviate the need for complicated and expensive culture protocols to induce naïve ESCs. Retroviruses such as MERV-L in mouse and HERV-H in human are therefore powerful tools to naturally select distinct populations of primitive pluripotent cells in order to study early development and offer new perspectives in reprogramming strategies and in the production of stem cell medicines. It is clear that retrotransposons impact on cell fate and reprogramming but how do they do this? Their distinct functions can be divided into three categories detailed below, which are (a) the recruitment of TFs to activate cellular genes, (b) the production of noncoding RNAs and finally, (c) potential roles of viral proteins and particles.

*(a) Retrotransposon DNAs bind core pluripotency factors in a species-specific manner* Retrotransposons have likely evolved to recruit activating TFs expressed early in development to ensure their propagation through the germ line and overall persistence in the genome. This has been beneficial to the host, providing us with a pool of regulatory sequences that can act as novel enhancers and promoters [20]. The creation of new TF binding sites within retrotransposons has occurred similarly in both mice and humans, although since many retrotransposon types and locations are distinct between these two species, this has led to unique gene regulatory circuits.

Several retrotransposons are abundantly expressed in human ESCs such as long interspersed nuclear element 1 (LINE-1) [21] and HERV-H [18]. This tissue-specificity is determined in part by TFs. For example, one LINE-1 family member, L1TD1 that has lost enzymatic activity is specifically expressed in ESCs because its promoter recruits NANOG, OCT4 and SOX2 [22]. HERV-H recruits the TF, LBP9 [19], and since this TF is essential for ground-state pluripotency [23], HERV-H, as one of its targets in human cells, is an integral component of naïve cells. LBP9 is a recent example that fits with a previous broad observation that core pluripotency TFs exert highly species-specific binding profiles. Indeed, it was established that up to 25% of pluripotency-associated TF binding sites were contributed from transposable elements, and that these TF-bound loci are lineage or species-specific because of the often random nature of the retrotransposition process [24]. Not all TFs are recruited to retrotransposon sequences and it appears that pluripotency associated TFs are enriched at these sites, although interestingly, the transcription insulator CTCF that is ubiquitously expressed also binds retrotransposons, but in this case B2 SINEs (short-interspersed nuclear elements) [25]. Certain classes of retrotransposons are enriched for particular TFs: for instance, OCT4/SOX2 binding is enriched on ERVK repeats in mouse and ERV1 in humans and TP53 is predominantly found on Mer61-type ERV1 repeats in human [26]. Sequence patterns that resemble binding site motifs of some TFs (ESR1, TP53, OCT4/SOX2, and CTCF) embedded within different transposable elements can therefore predispose them to becoming mammalian TF binding sites.

The majority of TF binding sites occur in the long terminal repeats (LTRs) of retrotransposons, which flank open reading frames (ORFs), or on UTRs of non-LTR retrotransposons such as LINE and SINE elements. LTRs and UTRs therefore serve as platforms for the recruitment of pluripotency-associated TFs and co-factors (such as chromatin modifiers) to moderate expression of cellular genes. The influence of retrotransposons on gene expression is significant because local epigenetic modifications can exert long-range effects by remodelling large chromatin domains and higher order chromatin structure, and LTR enhancers may loop to cellular genes in *cis* or even in *trans,* for example through CTCF [25]. In human and mouse ESCs, it was recently shown that LTR-derived enhancers affected numerous genes involved in chromatin organization, cell cycle and stemness [27]. We have also shown that repressed LTRs can become enhancers when they escape silencing, showing that silenced LTRs contain intrinsic enhancer activity and may act as temporal enhancers [28]. Similarly, LTRs also exert significant promoter effects that can be switched on during critical developmental stages. For instance, waves of stage-specific retrotransposon activation occur during pre-implantation embryo development. Indeed, by investigating the transcriptomes of human pre-implantation embryos, Goke and colleagues [29] have documented transient ERV activation taking place between the oocyte and blastocyst stages. Their analysis of different ERV families revealed that each stage of the embryo expresses distinct ERV classes, for example, HERVK14 is only expressed between the pronucleus and 4-cell stage, whereas THE1A is restricted to the 8-cell and Morula stages. Interestingly, both elements are not expressed in human ESCs, reinforcing the idea that cultured human ESCs only represent a snapshot of development and are primarily not naïve ESCs [29]. Another example of a stage-specific ERV is MERV-L [30], whose promoters drive expression of hundreds of 2-cell stage expressed genes [10]. While retrotransposons can act as promoters or enhancers for cellular genes, their enhancer function is particularly fascinating due to the recently documented rapid evolution of enhancer modules across species, which includes enhancers derived from retrotransposon sequences [31].

*(b) Non-coding RNAs derived from LTRs play a role in stem cell identity* Pluripotency TF binding to retrotransposons leads to transcription not only of cellular genes but also of retrotransposon RNAs, some of which are coding and many of which are short or long non-coding RNAs (lncRNAs) of largely unknown function. Interestingly, some non-coding RNA molecules are chimeric because they originate from splicing events between cellular and viral transcripts. Of particular interest, rare sub-populations of human ESCs (termed naïve cells, introduced above) have an active chromatin configuration at LTR7 sites in the genome (hypomethylated DNA, active histone marks and bound NANOG, OCT4, KLF4 and LBP9) and show elevated expression of their linked HERV-H transcripts, as well as of HERV-K transcripts, ([19, 27, 32] and see Figure 1b). High-throughput transcriptional profiling of mouse and human stem cells has revealed a large pool of species-specific chimeric and lncRNAs, including several pluripotency-associated lncRNAs, such as lin-ROR [33] and linc00458 [34].

Increased expression of some members of the HERV-H family is also observed in human induced pluripotent stem cells (iPSCs) [17, 35]. Depletion of some HERV-H expressed loci or of lin-ROR in human ESCs, using RNA interference, results in a drastic change in cellular morphology, with cells adopting a more differentiated phenotype (fibroblast-like) [32]. LncRNAs are implicated in many cellular processes including chromatin remodelling, control of promoter activity, X-chromosome inactivation, imprinting and nuclear import (reviewed in [36]). However, the specific role of most lncRNAs remains unclear. In the context of stem cells, retrotransposon-derived transcript expression levels closely mirrors the expression patterns of core pluripotency factors such as OCT4, NANOG and SOX2, suggesting that they might be essential to the pluripotent state. Importantly, HERV-H must be silenced to guarantee successful cell differentiation [34]. Reminiscent of pluripotent cells, LTR7-induced transcripts (including HERV-H lincRNA-RoR [33], LINE-1 [37] and HERV-K [35]) are transiently activated during reprogramming to iPSCs, indicating that their role in ESCs may parallel their role in restoring pluripotency in differentiated cells. However, they need to be subsequently repressed for successful reprogramming [17]. In general, high HERV-H RNA levels define naïve ESCs, concomitant with a complete loss of repressive chromatin marks such as condensed H3K27me3, whereas HERV-H is only lowly detected in primed ESCs. Of note, a similar enrichment of ERV derived transcripts has also been described in trophoblast stem cells and placenta [38].

However, what do HERV-H-lncRNAs do? Recent work has shown evidence that these RNA molecules can recruit transcriptional co-activators and other proteins into DNA-binding regulatory complexes. A specific function of long intergenic non-coding RNAs (lincRNA) was first demonstrated in human fibroblasts and their derivative iPSCs where a lincRNA specific microarray analysis was performed [33]. Additionally, RNA cross-linking experiments in human ESCs show that HERV-H-lncRNAs act as a scaffold unit that recruits the co-activators CBP, p300, MED6 and MED12, to enhancer regions [32]. These lncRNAs are also associated with OCT4, and are thought to play an essential role in LTR-specific enhancer activity (Figure 1b). For example, one of these co-activators is the histone acetyltransferase p300, which was showed to be essential for the recruitment of the NANOG/OCT4/SOX2 complex and regulates transcription via chromatin remodelling [39].

In sum, the involvement of retrotransposon lncRNAs in the control of pluripotency in early development and in reprogramming is a common mechanism in mammals, likely acting through RNA-recruited co-activators but operating via species-specific transposable elements.

*(c) Viral proteins, and particles that bud from embryos may function in development* Some of the first electron microscopy images of chicken embryos revealed mysterious virus-like particles (VLPs) of unknown function, which were mainly extracellular [40]. Likewise, in mouse embryos, ERVs, including of the IAP and MERV-L classes can be observed budding into the endoplasmic reticulum, particularly at the 2-cell stage [41]. The potential function of retroviral particles is unknown, although they may serve an antiviral role. In contrast, it is well established that certain retroviral proteins serve vital functions in reproduction and development. The best example of this is the *syncytin* family: *syncytins* 1 and 2 are essential placental genes derived from retroviral envelopes. These proteins emerged in mammals on at least six occasions independently and were retained each time by natural selection to carry out the same function. Syncytins are responsible for the formation of the syncytiotrophoblast, the multicellular element of the placenta responsible for nutrient exchange and shielding the embryo from the mother’s immune system [42].

Another documented example of retrotransposon protein expression is for LINE-1. It was recently demonstrated that mammals have evolved to use LINE-1 retrotransposon activity as a way to assess the quality of gametes. The massive loss of oocytes (two-thirds are lost in mice and around 80% in humans) that takes place during their maturation serves as a key quality-control checkpoint. For example, a recent study demonstrated that levels of the LINE-1 protein, L1ORF1p, which is essential for retrotransposition, acted as a marker to govern oocyte fate. Apoptosis is triggered only in oocytes with high L1ORF1p levels, ensuring that aberrant LINE-1 activation during epigenetic reprogramming of the genome remained as low as possible in the surviving oocytes and potential offspring [43]. An analogous mechanism likely exists in the male germ line. Conversely, the L1TD1 gene is an interesting example of a LINE-1 protein that has been positively selected in both primates and mice, due to beneficial roles it is thought to play in both genome defence and pluripotency [44]. Of note, active retrotransposition of LINE-1 has been reported in neural progenitor cells and brains of rodents and humans [45–48], although the potential function of this is unknown.

Although it is largely unknown how viral proteins and particles might contribute to genome defence and maintain pluripotent states, an exciting recent study on HERV-K provides new insight into these questions [49]. The authors reveal firstly that OCT4 drives expression of human-specific HERV-K proviruses by binding to their promoters (LTR5HS), leading to the production of GAG proteins and VLPs during early human development. Secondly, the HERV-K accessory protein, Rec binds to a subset of cellular mRNAs and can influence their translation, and finally HERV-K may serve to combat exogenous viral infections because it is shown to upregulate classical virus restriction factors such as IFITM1. Of note, data from this paper also suggests that HERV-K may be a more accurate marker of naive human ESCs than HERV-H because it is expressed in naive but not primed human ESCs.

## Part II: Retrotransposons are repressed in ESCs

### KAP1 retrotransposon repression shapes gene regulation

The very retrotransposons that are active and have been exapted to serve useful gene regulatory functions are often the same families that our genomes have evolved to repress. This is because these families contain elements with intact regulatory sequences that could interfere with gene expression and/or functional open reading frames that could lead to retrotransposition events. One example of this is MERV-L, which, as mentioned above, is highly abundant at the 2-cell stage of development (contributing to 3% of mRNAs) but repressed by the blastocyst stage [30].

Active retrotransposon families are targeted for epigenetic silencing early in development and during reprogramming [17, 35, 50]. One important repression pathway we and others have uncovered is the KAP1 (TRIM28) pathway that operates in ESCs and early embryos: KAP1 is recruited to repetitive sequences through site-specific krüppel-associated box domain-containing-zinc finger proteins (KRAB-ZFPs) and represses them through the histone methyltransferase ESET/SETDB1 [51–56] (and reviewed in [57, 58]), leading to their subsequent DNA methylation [59]. ERV silencing also leads to the repression of nearby genes due to the spreading of epigenetic marks, suggesting that this mechanism may have been co-opted for the fine-tuning of gene expression; certain genes are not switched off but maintained in a lowly expressed state in early development [28, 60, 61].

**Figure 2:**
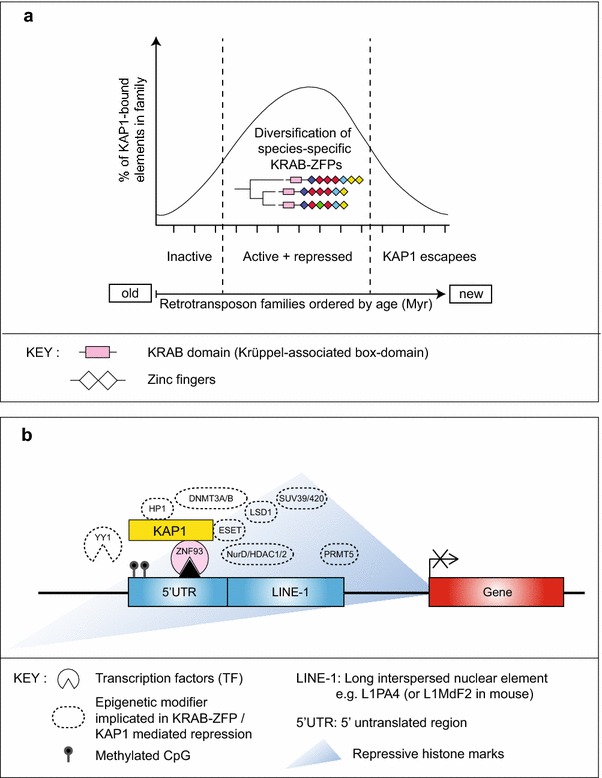
Adaptive evolution of retrotransposon repression in ESCs. **a** Co-evolution of retrotransposons and KRAB-ZFPs. **b** Mechanism of KAP1 repression of retrotransposons.

KAP1 repression is sequence-specific in vitro and in vivo [59] and retrotransposons that have more recently invaded the genome escape KAP1 through subtle changes in their nucleotide content, presumably because the KRAB-ZFP system has not yet adapted to repress them. This is true for both mouse (for example for the IAP class [53]) and human retrotransposons (LINE-1 [51]), although it is best illustrated with the LINE-1 family, due to the recent classification of LINE families based on their relative ages [62]. The most ancient LINE-1 families are neither KAP1-bound nor DNA methylated, presumably because they are dead by mutation, whereas the newer ones are KAP1-bound, repressed and highly DNA methylated, and finally the most recent families escape KAP1-repression, but they are regulated through DNA methylation, which may be deposited through one or more small RNA pathways ([51] and see Figure 2a). The KAP1-ERV repression pathway operates in mouse and human ESCs but is not required in mouse embryonic fibroblasts [53, 54], presumably because DNA methylation takes over as the dominant silencing mechanism later in development, a hypothesis we and others have provided evidence for [59, 63, 64]. Of interest, KAP1 repression of ERVs is still detected in mouse neural progenitor cells [65].

KAP1 repression of retroviruses was initially discovered in the context of murine leukaemia virus (MLV) [55], which led on from original observations that MLV was restricted in embryonic cells [66] through its primer binding site Pro (PBS-pro) sequence that binds proline tRNA [67, 68]. This sequence was later discovered to recruit KAP1 through the mouse KRAB-ZFP, Zfp809 [56]. MLV still serves as a practical model to explore the KAP1 repression pathway and it was recently uncovered that YY1 and EBP1 contribute to an MLV silencing complex [69–71], factors that may also repress ERVs with a PBS-pro and/or ERVs with unrelated PBS sites (reviewed in [72]). Elegant work has just revealed, through Zfp809 knockout mice and genetic and biochemical experiments in ESCs, that Zfp809 not only restricts exogenous MLV but also several ERVs that contain PBS-pro sequences, as predicted [73]. Strikingly, in Zfp809 knockout mice, disruption of silent chromatin marks normally established early in development at VL30-(virus-like 30) type PBS-pro ERVs leads to their overexpression in differentiated tissues, together with nearby genes. Of note, VL30 elements lack coding regions, illustrating how KAP1 represses not only coding but also non-coding ERVs that remain a threat because of their regulatory sequences. This new study, therefore, provides conclusive evidence that the KAP1/KRAB-ZFP pathway is necessary to repress retrotransposons and linked genes in vivo.

### Human retrotransposons are targeted by human-adapted KRAB-ZFPs

The finding that KAP1 represses multiple classes of ERVs (mainly IAPs and MERVK in mouse [53] or HERVK and LINE-1 in humans [54], most of which do not operate through a PBS-pro or even have mutated PBS sequences or no PBS sequence (LINE-1s), led to the concept that retrotransposons may recruit KAP1 through a multitude of different site-specific KRAB-ZFPs. This would ensure that even retrotransposons that cannot reverse transcribe are maintained inactive so as not to affect cellular genes through their potentially active enhancer/promoter sequences [28, 60]. This is supported by the diverse KRAB-ZFPs that our genomes encode, many of which are species-specific and rapidly evolving with largely unknown functions, which suggests their participation in genetic conflict with viral sequences that are also rapidly evolving [74–78]. One example of KRAB-ZFP adaptive evolution is the ZNF91 subfamily that has expanded across primate lineages [79].

However, while previously only a model, it has not been until now that exciting work has illustrated that indeed our genomes do encode a repertoire of KRAB-ZFPs adapted to recognize and target species-specific retrotransposons. Specifically, the human proteins ZNF91 and ZNF93 bind to and repress SVA and LINE-1 retrotransposons respectively, in the human genome [80]. SVA elements are a newly emerged retrotransposon class that invaded great ape genomes 18–25 million years (myr) ago. They are composite retrotransposons that contain an Alu-like fragment that is joined by a variable number tandem repeat (VNTR) domain to a SINE region that contains 3′LTR sequences similar to the ERV, HERV-K10 [81]. ZNF91 underwent structural changes between 8 and 12 myr ago to restrict SVAs, including the addition of seven zinc fingers. The authors nicely link structure to function showing that while macaque ZNF91 is unable to repress a human SVA reporter plasmid in mouse ESCs, (which do not express endogenous ZNF91), transfected human ZNF91 with its seven newly evolved zinc fingers induces strong SVA repression, as expected [80].

ZNF93 is another interesting example of a host-retrotransposon interaction, particularly because LINE-1s exert a unique pattern of evolution with a single L1PA subfamily active at one time in a genome before being replaced by a new subfamily, allowing their approximate ageing [62]. For example, in the human genome, L1PA4 (18 myr old) was replaced by L1PA3 (15.8 myr old), which was replaced by L1PA2 (7.6 myr old). KRAB-ZFP evolution relates to the activity of LINE-1 subfamilies because ZNF93 targets a sequence present in the L1PA4 UTRs and some L1PA3 UTRs but which is deleted in L1PA2 elements leading to their escape from ZNF93 repression in mouse ESCs when LINE-1 reporters and ZNFs are co-transfected [80]. ZNF93 underwent zinc finger deletions and other structural adaptations to repress human LINE-1s. As such, human ZNF93 but not macaque ZNF93 is able to repress an L1PA4 reporter construct in mouse ESCs. In the case of SVAs, nearby genes were also repressed through ZNF91, which supports previous findings that retrotransposon repression can lead to species-specific fine-tuning of gene circuits in vitro [28, 60] and in vivo [73]. Species-specificity is driven not only by LINEs and SVAs, some of which are distinct in the human genome but also by the TFs they recruit, which are also species-specific, as they have undergone adaptation between primate lineages. These new findings lead us to a summary model of KAP1 repression of retrotransposons, although the exact enzymes required are still unclear (See Figure 2b).

Of note, still only a handful of KRAB-ZFPs that recognise repetitive DNA have been characterized to date. This includes the two human KRAB-ZFPs discussed above and the mouse KRAB-ZFP Zfp809, already mentioned [55, 56, 73]. Apart from these, an additional mouse KRAB-ZFP, Zfp819 has been implicated in modulating expression of IAP ERVs and LINEs through an unknown sequence, which impacts on the balance between pluripotency and differentiation [82] and the new mouse KRAB-ZFP *Gm6871* that was previously only a predicted gene, targets a subset of LINEs (mainly of the L1MdF2 family), again through a 5′UTR sequence [51]. A mouse KRAB-ZFP *Ssm1b* has also been implicated in DNA methylation of foreign DNA [83]. Many questions persist concerning how KRAB-ZFPs exert their functions, their patterns of evolution, how many of them recognise repetitive sequences, where and when they act, how they impact on cellular genes and how they relate to disease settings. However, a recent paper provides evidence that most KRAB-ZFPs may target repetitive sequences since 16 out of 18 human KRAB-ZFPs sampled at random bound repeated elements, including LINE-1, HERVs and SVAs, as determined by chromatin immunoprecipitation [84]. Of note, the authors also used high throughput binding data to create an improved ZFP recognition code predictor.

### Other retrotransposon repression pathways

While a discussion of all other potential retrotransposon repression pathways acting within ESCs is beyond the scope of this review, we mention a few of the main ones below and refer to other reviews [72, 85]. These can be divided into transcriptional repression pathways, which together with KAP1 act as the first line of defence against retrotransposons, and post-transcriptional repression pathways that are crucial at later stages of the retrotransposon life cycle to prevent new retrotransposition events.

Transcriptional repression pathways involving histone deacetylases and histone methyltransferases/demethylases are fundamental to retrotransposon repression in ESCs because in these cells, there is a layer of repression additional to DNA methylation, highlighted by the finding that triple knockout of DNMT1, DNMT3a and DNMT3b is not sufficient to significantly reactivate retrotransposons such as IAPs [63, 86]. DNA methylation-independent mechanisms of repression are presumably required in development in the face of global re-setting of the epigenome. Key enzymes implicated in retrotransposon repression in ESCs include HDACs [87, 88], ESET/SETDB1 [52, 59, 89], which likely has KAP1-independent as well as dependent targets, LSD1/KDM1a [90], G9a [91], Suv420H1/2 that mediates H4K20me3 [28, 52], polycomb complexes [92] and Suv39h [93]. Other less studied enzymes such as the arginine methyltransferase PRMT5 may also play a role, since it has recently been uncovered to interact with KAP1 [94] and repress some retrotransposons in primordial germ cells and preimplantation embryos during DNA methylation reprogramming [95]. The role of PRMT5 in retrotransposon repression was also assessed in *prmt5* deleted and control 2i- cultured ESCs (see ESC culture protocols in part I above), which display DNA hypomethylation, but only 1.8-fold of IAP-Gag upregulation was observed in this context, perhaps due to PRMT5 compensation by PRMT7. Other co-factors that have been implicated in retrotransposon transcriptional repression include HP1 family members that interact with KAP1 [96–98] and may be involved in long-range repression [99, 100], DNMT3L [101, 102], hnRNPK and MCAF1 [103], REX1 [104, 105] and SIRT6 [106]. Exactly which enzymes are directed to certain retrotransposons and how and when KAP1 is involved is unknown. It is possible that non-coding RNAs play a targeting role in a similar way to how they tether co-activators to HERV-H (see part I above).

Post-transcriptional repression pathways include intrinsic factors that block later stages of the retrotransposon lifecycle such as SAMHD1 ([107] and see [108] for a review). A multitude of small RNA pathways are crucial to retrotransposon regulation in development and the intricate details of these pathways are only now being unravelled. Some small RNAs like piRNAs (small noncoding piwi-interacting RNAs) can even induce the silent histone mark H3K9me3 and de novo DNA methylation at least in the germ line, adding to transcriptional silencing [50, 109–111] and the piRNA pathway may play a role in ESCs [112]. Interestingly, small RNAs derived from LINE-1 have been implicated in transcriptional activation of LINE-1 at the 2-cell stage of embryo development [113], whereas in mouse ESCs, there is a role for small RNAs in LINE-1 restriction because there is an increase in LINE-1 transcripts in Dicer knockout ESCs that is rescued by ectopic Dicer expression [114]. Still much is unknown about small RNA transposon silencing in ESCs, particularly as small RNAs detected (in this case from LINE-1) include both Dicer-dependent and independent classes [114].

## Discussion and perspectives

As discussed, retrotransposons can confer regulatory complexity to gene networks early in development and in ESCs by serving as enhancers and/or promoters of key developmental genes. They provide new regulatory sequences that have been integrated into novel gene networks and they are particularly important in the maintenance of the naïve pluripotent cell state, likely through their adaptation to bind TFs expressed early in development (see [72, 85, 115, 116] for additional reviews on this topic).

Many questions remain about the extent to which a family of retrotransposons can be repressed or activated in certain tissues or at particular developmental stages, and which factors coordinate switches in activation status. One interesting perspective is that the very KRAB-ZFP pathways that repress retrotransposons may actually only induce temporal or tissue-specific repression, and there is some evidence for KRAB-ZFPs even being activators in some cases [76]. It would make sense for the host to evolve to restrict retrotransposons in situations where they pose heritable threats to the germ line such as in germ cells or early embryos where many KRAB-ZFPs are enriched. This may have led to retrotransposons becoming ideal lineage-specific enhancers that get switched on during later stages of development, a hypothesis worth exploring. Another interesting perspective is that since KRAB-ZFP gene clusters are heavily intermingled with retrotransposons, some of them may have been reverse transcribed along with retrotransposons, which may have contributed to their rapid evolution and increase in copy number.

In summary, retrotransposons are, unlike classical viruses, essential to the evolution of our genomes and have contributed to genome plasticity and the creation of new genes. We can use them as tools to direct cell fate and reprogramming but studying their intricate pathways of expression is necessary to understand gene regulation in development, and to develop safe stem cell medicines. Research into this topic is particularly relevant to understanding and treating genetic diseases and cancers in which retrotransposons have been implicated.
